# Low-Dose Oxygen Enhances Macrophage-Derived Bacterial Clearance following Cigarette Smoke Exposure

**DOI:** 10.1155/2016/1280347

**Published:** 2016-06-14

**Authors:** William G. Bain, Ashutosh Tripathi, Pooja Mandke, Jonathan H. Gans, Franco R. D'Alessio, Venkataramana K. Sidhaye, Neil R. Aggarwal

**Affiliations:** ^1^Division of Pulmonary and Critical Care Medicine, Johns Hopkins University School of Medicine, Baltimore, MD 21224, USA; ^2^Division of Pulmonary, Allergy, and Critical Care Medicine, University of Pittsburgh School of Medicine, Pittsburgh, PA 15213, USA

## Abstract

*Background.* Chronic obstructive pulmonary disease (COPD) is a common, smoking-related lung disease. Patients with COPD frequently suffer disease exacerbations induced by bacterial respiratory infections, suggestive of impaired innate immunity. Low-dose oxygen is a mainstay of therapy during COPD exacerbations; yet we understand little about whether oxygen can modulate the effects of cigarette smoke on lung immunity.* Methods.* Wild-type mice were exposed to cigarette smoke for 5 weeks, followed by intratracheal instillation of* Pseudomonas aeruginosa* (PAO1) and 21% or 35–40% oxygen. After two days, lungs were harvested for PAO1 CFUs, and bronchoalveolar fluid was sampled for inflammatory markers. In culture, macrophages were exposed to cigarette smoke and oxygen (40%) for 24 hours and then incubated with PAO1, followed by quantification of bacterial phagocytosis and inflammatory markers.* Results.* Mice exposed to 35–40% oxygen after cigarette smoke and PAO1 had improved survival and reduced lung CFUs and inflammation. Macrophages from these mice expressed less TNF-*α* and more scavenger receptors. In culture, macrophages exposed to cigarette smoke and oxygen also demonstrated decreased TNF-*α* secretion and enhanced phagocytosis of PAO1 bacteria.* Conclusions.* Our findings demonstrate a novel, protective role for low-dose oxygen following cigarette smoke and bacteria exposure that may be mediated by enhanced macrophage phagocytosis.

## 1. Background

Chronic obstructive pulmonary disease (COPD) is primarily a smoking-related lung disease and afflicts more than 200 million people worldwide. Pathologically, COPD is characterized by chronic bronchitis or emphysema. In chronic bronchitis, airway inflammation leads to increased mucus production and reduced mucociliary clearance, causing bronchoconstriction and airflow limitation. Emphysema is hallmarked by destruction of lung parenchyma. Patients with COPD frequently suffer disease exacerbations often induced by bacterial or viral respiratory infections, suggestive of impaired innate immunity. During disease exacerbation, supplemental oxygen is a mainstay of therapy for COPD patients. Although there is clear benefit to continuous low-level oxygen therapy in chronic, stable COPD disease [1, 2], excessive amounts of nontitrated oxygen may in fact be harmful during COPD exacerbation [3, 4]. Therefore, how oxygen modulates the effects of cigarette smoke on lung immunity may be relevant for patients with COPD exacerbation and other smoke-induced lung diseases.

The immunomodulatory effects of moderate and high levels of oxygen exposure (FiO_2_ 0.6–1.0) have been well described in experimental models. Mice exposed to four days of 95% oxygen had increased mortality with impaired macrophage phagocytosis in a* Klebsiella pneumoniae* model [5]. 60% oxygen exposure shortly following LPS-induced lung injury markedly exacerbated lung inflammation in part mediated by macrophage-induced recruitment of alveolar neutrophils [6]. Although higher levels of oxygen appear to impair lung immunity, there is limited data as to how lower levels of oxygen supplementation (FiO_2_ 0.30–0.4) can modulate lung immunity, particularly with coexisting cigarette smoke exposure.

Macrophages are prominent resident cells of the alveolar space and are critical for regulation of immune responses in the lung [7–9]. With smoke-induced COPD, macrophage numbers can increase 5- to 10-fold in the lungs and alveolar space [10, 11] and can correlate with disease severity [12], although they do not conform to the classic M1/M2 dichotomy [13]. Alveolar macrophage dysfunction induced by cigarette smoke exposure leads to excess oxidative stress and may contribute to higher bacterial colonization and increased susceptibility to exacerbations. Alveolar macrophage phenotype and function can be modulated by exposure to varying levels of supplemental oxygen [5, 14].

As a model to help understanding oxygen effects on the lung immune response to bacteria subsequent to cigarette smoke exposure, we challenged mice or macrophages in isolation with subacute durations of cigarette smoke (CS) followed by exposure to low-dose oxygen and* Pseudomonas aeruginosa* bacteria. We chose a shorter cigarette smoke exposure to focus on the effects CS and oxygen-induced changes in inflammatory cell populations [15], similar to bronchitis, and without emphysema-like changes in lung architecture. We present here the results of our findings.

## 2. Methods

### 2.1. Animal Use, Care, Smoke, Bacteria, and Oxygen Exposure

C57Bl/6 mice were purchased (Jackson Labs, Bar Harbor, ME) and housed at the Johns Hopkins University Asthma and Allergy Center. Experiments were conducted under a protocol approved by the Johns Hopkins Animal Care and Use Committee. C57Bl/6 mice were exposed in the Johns Hopkins smoke exposure core. Mice were exposed to cigarette smoke 5 hours/day, 5 days/week for 5 weeks, by burning 3R4F reference cigarettes (2.45 mg nicotine/cigarette; Tobacco Research Institute, University of Kentucky) using a smoking machine (Model TE-10, Teague Enterprises).

For specified groups, the morning after a 5-week cigarette smoke (CS) exposure, we instilled* Pseudomonas aeruginosa* (PAO1, ATCC, Manassas, VA) (3 × 10^6^ CFUs, in 50 *μ*L PBS) or vehicle control (PBS) via an intratracheal (i.t.) route through a 20-gauge endotracheal catheter as before [16]. Mice were anesthetized with intraperitoneal ketamine/acetylpromazine (100/2.5 *μ*g/g) prior to exposure of the trachea. Within 2 hours of instillation of i.t. PAO1 or vehicle control, mice were exposed to 35–40% oxygen or 21% oxygen for up to five days. For oxygen exposure, mice were placed in customized and sealed cages with ad libitum food and water. 35–40% oxygen was achieved with a mixture of air and medical grade oxygen (Roberts Oxygen, Rockville, MD) at adjustable flow rates and constant pressure, with continuous measurements via an oxygen analyzer with a feedback loop to automatically adjust oxygen concentrations (model 65, Advanced Micro Instruments, Huntington Beach, CA). Oxygen exposure was uninterrupted except for 5 min every other day for cage cleaning.

### 2.2. Animal Harvesting and Bronchoalveolar Lavage (BAL)

Mice were harvested after 5 weeks of cigarette smoke exposure (or air exposure as a control) and 3 days of 35–40% oxygen (or 21% oxygen as a control). In addition, following 5 weeks of CS exposure (or air exposure), designated mice were exposed to i.t. PAO1 (or vehicle control) and 2 days of 35–40% oxygen (or 21% oxygen as control) until sacrifice and harvest of lungs for assessment of CFUs and inflammatory parameters or up to 5 days for assessment of mortality. Mice were anesthetized with intraperitoneal ketamine/acetylpromazine (150/13.5 mg/kg) prior to harvest and killed by exsanguination from the inferior vena cava. The lungs were perfused free of blood with 1 mL of phosphate-buffered saline (PBS). BAL was obtained by cannulating the trachea with a 20-gauge catheter. The right lung was lavaged with two aliquots of 0.7 mL calcium-free PBS. For quantitative measures of bacteria, whole lungs were homogenized without prior lavage, and the lysates were diluted in PBS and streaked on agar plates. After 24 hours at 37°C, colonies were counted.

### 2.3. BAL Processing and Analysis

BAL fluid was centrifuged (700 ×g, 10 min at 4°C), and cell-free supernatants were stored at −80°C. The cell pellet was diluted in PBS, and the total cell number was counted with a hemocytometer after staining with trypan blue. Cell populations were determined by counting 300 cells/sample, and a percentage was calculated based on a minimum of three mice per group. Total protein was measured in the cell-free supernatant using the Lowry method [17].

### 2.4. Cell Culture

MH-S alveolar mouse macrophage cells were obtained from ATCC (Manassas, VA). Cells from passages 4–10 were maintained in Dulbecco's modified Eagle's media with 10% fetal bovine serum, 1% L-glutamine, and 1% penicillin/streptomycin mixture at 37°C in 5% CO_2_. Prior to experiments, cells were scraped from culture, collected, and centrifuged at 300 RCF for 5 minutes and then counted via trypan blue exclusion. MH-S cells were added in media to 0.4 nM-tissue culture PET-membrane inserts (Falcon) and placed in media and in six-well plates (Falcon). Cells were maintained at 37°C in 5% CO_2_ between 2 and 24 hours to allow adherence to tissue culture inserts prior to experiments, which was verified by light microscopy. In experiments utilizing serum-free media, media was removed from inserts and wells.

### 2.5. *In Vitro* Smoke, Oxygen, and Bacteria Exposure

MH-S alveolar macrophage cells cultured on inserts were placed into a Vitrocell chamber (Waldkirch, Germany) for exposure to whole cigarette smoke (2 cigarettes, 7 min/cigarette) or room air sham for 2 exposures over 24 hours [15]. Cells were treated with 40% O_2_/5% CO_2_ or 21% O_2_/5% CO_2_ (control) between CS exposures. Cell-free supernatants were collected after the 24-hour CS exposure.

PAO1-GFP was obtained from ATCC. Bacteria was harvested from agar plates following 24-hour incubation at 37°C, inoculated in LB broth, and then incubated at 37°C and 200 RPM until in log phase of growth. Bacteria were then diluted to OD = 0.1 at 600 nm, approximating 1 × 10^8^ CFU/mL of live bacteria. Bacteria were washed in PBS and used immediately. MH-S cells were incubated with GFP-labeled* Pseudomonas aeruginosa* (PAO1-GFP) in phosphate-buffered saline (PBS) for 3 hours, during which time exposure to 40% oxygen/5% CO_2_ (or control, 21% O_2_/5% CO_2_) was continued.

### 2.6. Macrophage Bacteria (PAO1) Phagocytosis

After 3 hours of incubation, the media were then removed and plated to determine extracellular PAO1-GFP CFU counts or collected for quantification of cytokines. After removal of PAO1-GFP inoculum, the MH-S cells on inserts were washed in warm PBS, treated with gentamicin (30–60 minutes) to kill adherent bacteria, and then washed twice in warm PBS and scraped from the inserts into detergent solution (1% Triton-X) that was plated for intracellular CFU counts using serial dilution.

### 2.7. Analysis of Cytokines

Using cell-free BAL fluid from mice or supernatants from cell culture, cytokine analysis of TNF-*α* and IL-6 was performed by standard ELISA kits following the manufacturer's recommendations (R&D Systems, Minneapolis, MN). All samples were run in duplicate.

### 2.8. Flow Cytometry (FACS)

For surface staining, primary lung cells or MH-S cells were incubated with Fc Block-2.4G2 (BD Pharmingen) Ab to block Fcg III/IIRs before staining with a specific Ab. The following antibodies were purchased from BD Pharmingen (San Diego, CA) and BioLegend (San Diego, CA): anti-Gr1-BV570, anti-CD11b-PETR, anti-CD86-BV421, anti-MMR-Ax647, anti-Dectin-1-Ax700, and anti-F4/80-allophycocyanin-Cy7, along with relevant isotype antibodies. The FITC/Ax488 channel was left open for PAO1-GFP. For intracellular staining of cytokines, cells were isolated and resuspended (0.5 × 10^6^ cells/mL) in RPMI 1640/FCS/penicillin/streptomycin/Golgi Plug (unstimulated) or with additional leukocyte activation mixture (BD Biosciences, San Jose, CA; PMA + ionomycin + brefeldin A; 2 mL/mL, stimulated, to enhance intracellular cytokine signal) for 4 hrs. Live-dead discrimination was performed with Fixable UV-Excitable Blue Dead Cell Stain (Invitrogen). Cells were Fc blocked; surface stained for macrophage, neutrophil, and lymphocyte markers; and fixed/permeabilized (Cytofix/Cytoperm, BD Pharmingen, San Jose, CA) and intracellularly stained × 30 min for cytokines including anti-TNF-*α*-PerCP. Monocytes, alveolar macrophages, neutrophils, and lymphocytes were gated with characteristic forward scatter/side scatter using a FACSAria instrument, CellDiva for data acquisition (BD Biosciences, San Jose, CA), and FlowJo for analysis (Tree Star, San Carlos, CA).

### 2.9. Statistical Analysis

Analysis was performed using GraphPad Prism 6.0 (La Jolla, CA) software. Student's *t*-test was used for comparisons between two variables with significance determined using the Holm-Sidak method. Multiple comparisons were performed using ordinary one-way ANOVA with Bonferroni or Tukey's correction for multiple comparisons. Survival analysis was performed using Kaplan-Meier curve with Mantel-Wilcox test. *p* < 0.05 was used as a cut-off to determine statistical significance.

## 3. Results

### 3.1. Low-Dose Oxygen after Subacute Cigarette Smoke Exposure Does Not Alter Lung Inflammation

**Figure 1:**
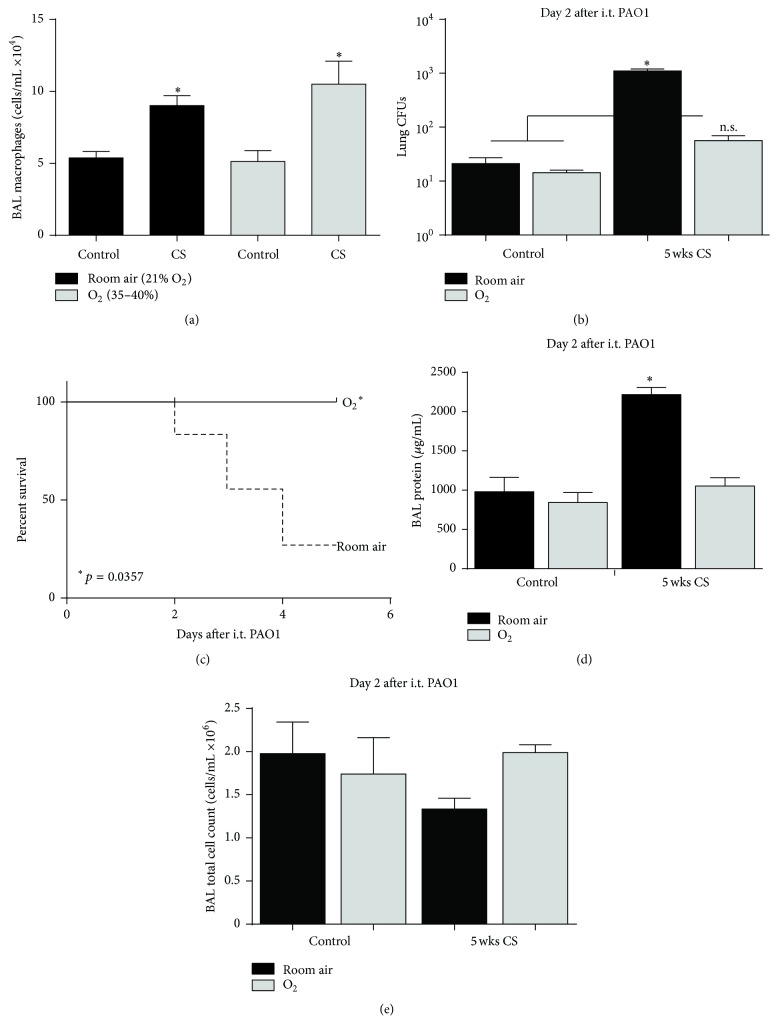
Low-dose oxygen is beneficial in CS- and PAO1-exposed mice. (a) Macrophages recovered by BAL from mice exposed to 5 weeks of cigarette smoke followed by 35–40% oxygen or room air for an additional 3 days (*n* = 4-5, ^*∗*^ *p* < 0.05 by one-way ANOVA). (b–d) Following 5-week CS or room air exposure, mice were exposed to PAO1 bacteria by intratracheal (i.t.) injection and either low-dose oxygen (35–40%) or room air (control), followed by up to 5 days of oxygen or room air exposure. (b) At day 2 after PAO1 exposure, mice were harvested and lung CFUs were quantified (*n* = 2-3, ^*∗*^ *p* < 0.001 against all other groups by one-way ANOVA, and n.s. compared to both control groups). (c) Among CS-exposed mice, Kaplan-Meier survival curve following i.t. PAO1 exposure (*n* = 6-7 mice, *p* = 0.0357 by log-rank Mantel-Cox). (d) Following bronchoalveolar lavage (BAL) on day 2 after PAO1 exposure, total protein was quantified (*n* = 2–4, ^*∗*^ *p* < 0.01 against all other groups by one-way ANOVA). (e) Following day 2 BAL, total cell count was quantified (*n* = 2–4).

The* in vivo* model consisted of wild-type C57Bl/6 mice exposure to 5 weeks of cigarette smoke via chamber as before [15]—controls were age-matched and exposed to room air also via chamber. We have shown that a subacute duration (4–6 weeks) of CS exposure was associated with increased alveolar epithelial permeability and increased accumulation of inflammatory cells in the alveolar space but did not induce changes in lung architecture [15]. In the current study, we observed a twofold increase in alveolar macrophages recovered by bronchoalveolar lavage (BAL) from cigarette-smoke-exposed mice compared to sham-exposed controls (Figure 1(a)). The addition of 3 days of continuous 35–40% oxygen exposure did not change the BAL macrophage count.

### 3.2. Low-Dose Supplemental Oxygen Promotes Clearance of Bacteria and Reduces Lung Injury in CS-Exposed Mice

CS- or air-exposed mice were exposed to* Pseudomonas aeruginosa* (i.t. PAO1, 3 × 10^6^ CFUs) followed by low-dose oxygen (35–40% O_2_) or control (21% O_2_, room air) for 2 days. Compared to air-exposed controls, smoke-exposed mice had increased PAO1 CFUs recovered from the lung, as others have also shown [18]. However, exposure to 35–40% oxygen (CS + O_2_) resulted in a significant decrease in PAO1 CFUs recovered from the whole lung compared to smoke-exposed mice exposed to room air (CS + room air) (Figure 1(b)). Low-dose supplemental oxygen also appeared to reduce bacteremia in CS-exposed mice as the CS + O_2_ mice had no evidence of bacteremia compared to ~500 PAO1 CFUs recovered from the blood of mice exposed to room air. Furthermore, between groups of CS-exposed mice, mice that received 35–40% oxygen for up to 5 days after PAO1 exposure had significantly reduced mortality compared to control (room air) exposure for the same period (Figure 1(c), *p* = 0.0357 by Mantel-Cox). There was no mortality after i.t. PAO1 in either non-CS-exposed group of mice (not shown).

To determine whether the benefits on bacterial clearance translated to other relevant endpoints, we also quantified lung injury parameters. At day 2 after i.t. PAO1, BAL protein was significantly increased in CS-exposed mice treated with 21% oxygen (CS + room air) compared to non-CS-exposed mice (Figure 1(d)). However, exposure to supplemental oxygen after i.t. PAO1 (CS + O_2_) reduced BAL protein to levels observed in non-CS-exposed mice. In contrast, the BAL total cell count at day 2 was not different between groups irrespective of CS or oxygen exposure (Figure 1(e)). Therefore, these data suggest that exposing mice to 35–40% oxygen following cigarette smoke and PAO1 exposure markedly improves bacterial clearance to improve survival, with some associated changes in lung injury parameters.

### 3.3. Low-Dose Oxygen Modulates Inflammation and Lung Macrophages in CS- and Bacteria-Exposed Mice

**Figure 2:**
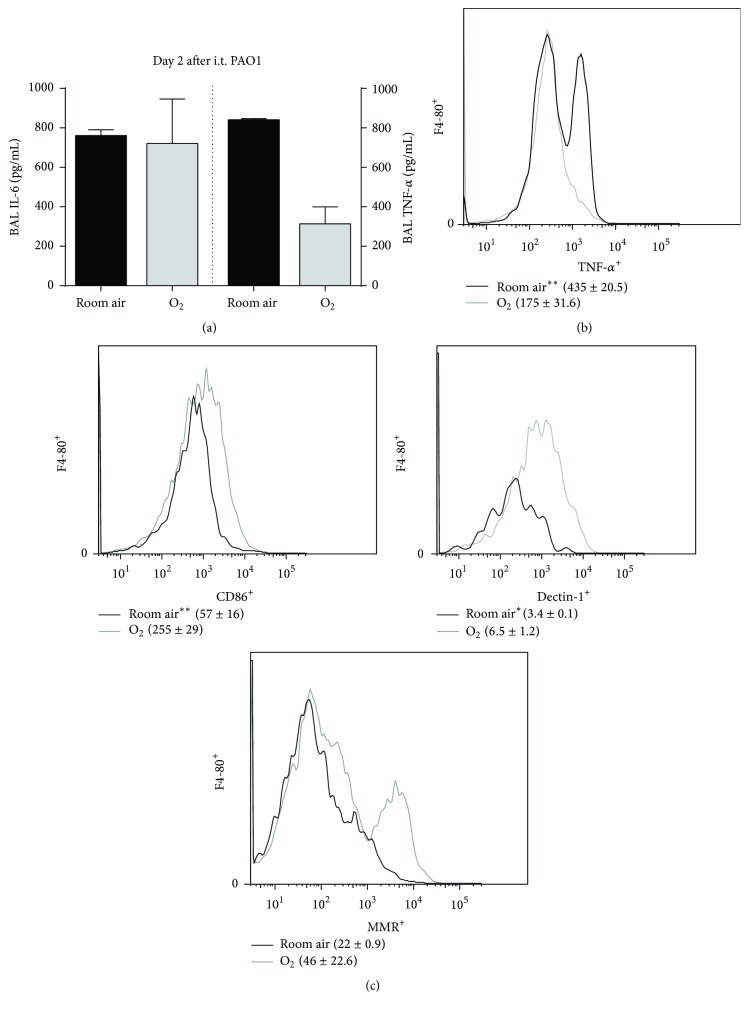
Oxygen alters the inflammatory profile and alveolar macrophage phenotype in CS and i.t. PAO1-exposed mice. (a) BAL cytokines were quantified following 2 days of PAO1 exposure in CS-exposed mice (*n* = 2–4). (b) Representative histogram of the mean fluorescent intensity (MFI) of expression of intracellular TNF-*α* using FACS among BAL F4-80^+^ macrophages (^*∗∗*^ *p* < 0.01 by unpaired *t*-test; MFI ± SD, *n* = 3). (c) Representative histogram of the MFI of the surface markers CD86, Dectin-1, and MMR on BAL F4-80^+^ macrophages (^*∗*^ *p* < 0.05, ^*∗∗*^ *p* < 0.01 by unpaired *t*-test; MFI ± SD, *n* = 3).

With the significant mortality benefit in O_2_-exposed mice following CS and i.t. PAO1 exposure, we measured BAL cytokine and cellular profiles to assess for other phenotypic differences (Figure 2). The addition of 35–40% O_2_ to CS and PAO1-exposed mice did not change BAL IL-6 levels but appeared to reduce BAL TNF-*α* at day 2 after i.t. PAO1 (Figure 2(a)). In addition, 35–40% oxygen exposure did not change the percentage of alveolar neutrophils (Gr1^+^) among CS- and PAO1-exposed mice at day 2 after i.t. PAO1 (not shown).

To further investigate how 35–40% oxygen exposure may induce macrophage-specific effects in CS- and bacteria-exposed mice, we used FACS analysis to measure TNF-*α* expression in macrophages as well as expression of macrophage surface receptors. After gating on a similar percentage and number of macrophages (F4-80^+^) between groups, mice exposed to low-dose oxygen had significantly decreased expression of TNF-*α* by FACS compared to room air exposure (Figure 2(b)). CD86, a proinflammatory M1 macrophage marker involved in cellular cosignaling [19], was significantly increased on macrophages derived from oxygen-exposed mice following CS and i.t. PAO1 exposure (Figure 2(c)). Furthermore, low-dose oxygen following CS and i.t. PAO1 exposure significantly increased expression of Dectin-1 and trended towards increased expression of mannose receptor, both M2, anti-inflammatory markers involved in phagocytosis. Collectively, these data suggest that low-dose oxygen modulates proinflammatory cytokine production and upregulates expression of macrophage receptors that may be important for bacterial clearance.

### 3.4. 40% Oxygen Exposure Enhances Bacterial Clearance by CS-Exposed Macrophages in Culture

**Figure 3:**
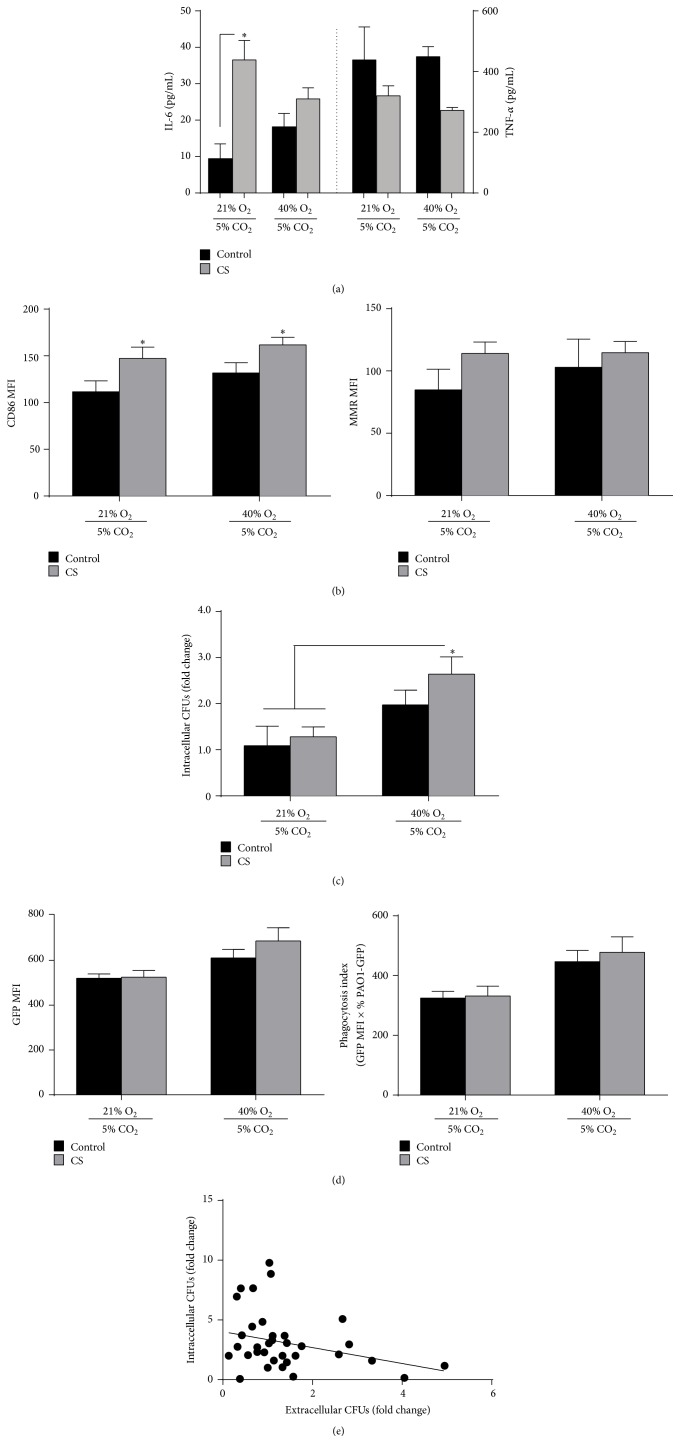
40% oxygen promotes bacterial clearance by CS-exposed macrophages. MH-S macrophages in cell culture were exposed to cigarette smoke (CS) or air (control) and 21% or 40% oxygen exposure for a total of 24 hours. (a) We quantified cytokines IL-6 and TNF-*α* in the cell-free media following the specified CS and oxygen exposure (*n* = 3, mean ± SEM, ^*∗*^ *p* < 0.05 by one-way ANOVA). (b) Using FACS, we measured CD86 and MMR MFI on CD11b^+^ cells prior to PAO1-GFP exposure (MFI ± SEM, *n* = 3, ^*∗*^ *p* < 0.05 compared to the control + 21% O_2_ group by one-way ANOVA with Bonferroni correction). (c) Following 24 hours of CS and oxygen exposure (or controls), we incubated cells with PAO1-GFP for 3 hours. Intracellular CFUs were expressed as a fold change compared to the control + 21% O_2_ group (mean ± SEM, *n* = 9, ^*∗*^ *p* < 0.05 compared to control + 21% O_2_ and CS + 21% O_2_ groups by one-way ANOVA with Bonferroni correction). (d) Using FACS following PAO1-GFP exposure, we quantified the MFI of GFP expression among MH-S cells (left) as well as the phagocytosis index (GFP MFI ×  %  MH-S cells with GFP signal). (e) Following PAO1-GFP exposure, we performed nonlinear regression of best fit across all data points to demonstrate the relationship of intracellular and extracellular CFU counts (each dot represents a single well, *n* = 8-9 for each group).

To further understand macrophage-specific effects induced by oxygen exposure, we adapted our* in vivo* model for cell culture. After 24 hours of exposure of MH-S alveolar macrophages to CS (or control) and 40% O_2_ (or 21% O_2_), IL-6 and TNF-*α* cytokines were quantified in the cell-free media, and macrophage marker expression was evaluated by FACS. CS exposure significantly increased IL-6 in the cell-free media compared to control (air) exposure (Figure 3(a)) but did not induce a difference in TNF-*α* at 24 hours, consistent with prior work [20]. The addition of 40% oxygen did not significantly modify IL-6 or TNF-*α* secretion. CS exposure significantly increased CD86 expression compared to air (control) (Figure 3(b)), but 40% oxygen did not further augment CD86 expression. In addition, CS also appeared to increase MMR expression on MH-S cells.

Following 24 hours of CS (or control) exposure, MH-S cells were incubated with* P. aeruginosa* (PAO1) with concurrent exposure to 40% oxygen (or 21% oxygen). After 3 hours, we quantified intracellular PAO1 CFUs. Exposure to 40% oxygen after CS and PAO1 resulted in a significant, greater than twofold increase in intracellular PAO1 by CFU counts when compared to air or CS-exposed groups that did not receive 40% oxygen (Figure 3(c)). Because PAO1 was GFP-tagged, we were also able to quantify the association of bacteria with macrophages using FACS (Figure 3(d)). Similar to the pattern observed with intracellular CFU counts, the addition of 40% oxygen exposure appeared to increase the GFP signal associated with MH-S macrophages both by mean fluorescence intensity (MFI) among GFP-positive macrophages, as well as the phagocytosis index (GFP MFI ×  % PAO1-GFP^+^) [5].

As a potential confounding factor, oxygen levels have been shown to influence the growth of* P. aeruginosa* [21]. To address this possibility, we quantified CFUs among extracellular bacteria not adherent to or phagocytosed by macrophages. Among all 4 exposure groups, we observed a strong inverse correlation (*R* ^2^ = 0.085) of extracellular CFUs with intracellular CFUs from the same well of MH-S cells (Figure 3(e)), suggesting that differences in intracellular bacteria were not attributable to oxygen-induced differences in bacterial growth in the media. Collectively, these data demonstrate that isolated macrophages exposed to cigarette smoke can augment bacterial clearance when treated with low-dose supplemental oxygen.

### 3.5. 40% Oxygen Modulates the Inflammatory Profile of CS-Exposed Macrophages in Culture

**Figure 4:**
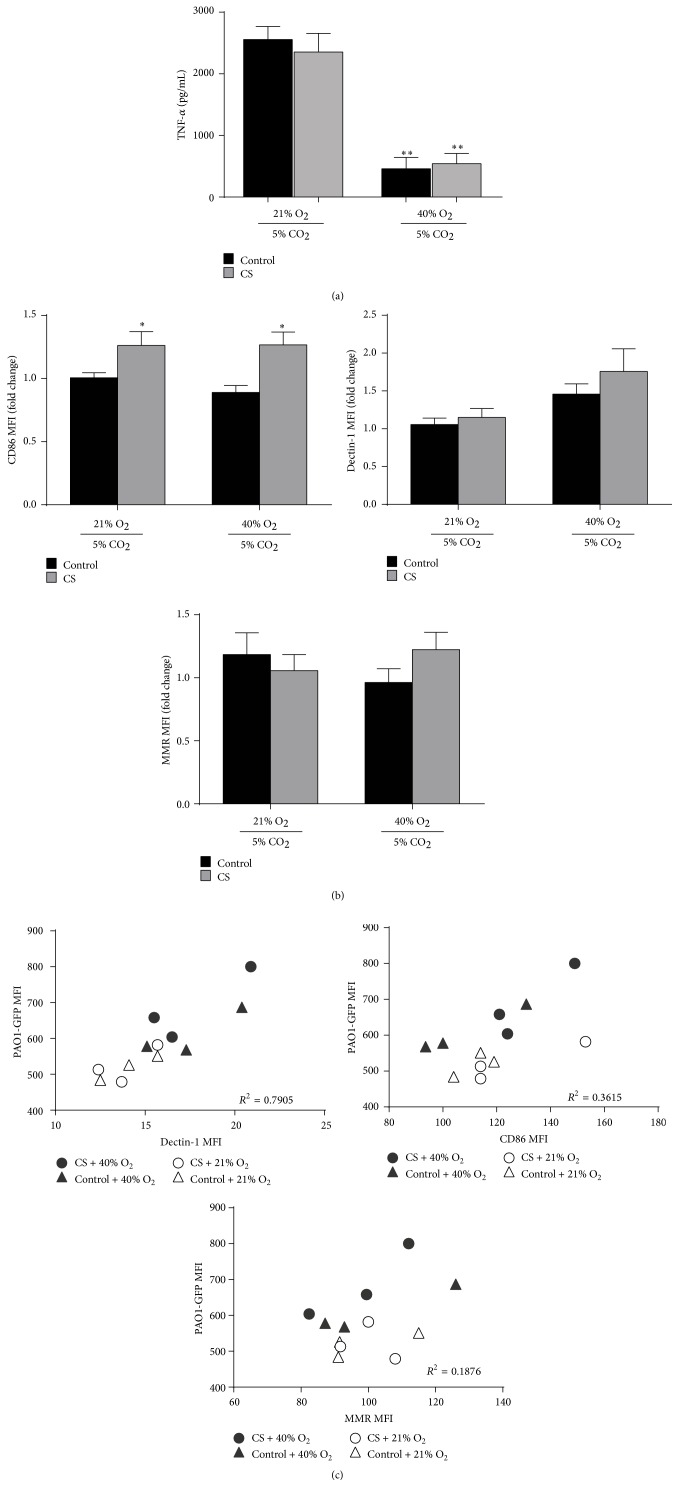
Oxygen modulation of macrophage phenotype may support PAO1 phagocytosis. (a) Following* in vitro* CS, oxygen, and PAO1 exposure, we measured TNF-*α* in the supernatant (*n* = 3, mean ± SEM, ^*∗∗*^ *p* < 0.01 by one-way ANOVA compared to both 21% oxygen-exposed groups). (b) Following* in vitro* CS, oxygen, and PAO1-GFP exposure, we measured the MFI of cell surface expression of macrophage markers CD86, Dectin-1, and MMR using flow cytometry and expressed values normalized to control across trials (mean ± SEM, *n* = 6, ^*∗*^ *p* < 0.05 compared to control + 40% O_2_ group by one-way ANOVA with Bonferroni correction). (c) Using FACS, we demonstrate scatter plots of macrophage GFP MFI expression (to denote PAO1 macrophage binding or engulfment) with macrophage MFI expression for Dectin-1 (*R* ^2^ = 0.7905), CD86 (*R* ^2^ = 0.3615), and MMR (*R* ^2^ = 0.1876) surface markers.

We also examined whether oxygen-enhanced bacterial phagocytosis by MH-S cells was associated with changes in its inflammatory profile by measuring selected proinflammatory cytokines and macrophage M1/M2 marker expression following PAO1 exposure. Although IL-6 was not different between groups (not shown), the addition of 40% oxygen to either CS or control-exposed MH-S cells significantly reduced TNF-*α* levels (Figure 4(a)). Expression of the M1 marker CD86 was increased on MH-S following CS and PAO1 exposure; the addition of 40% oxygen did not further augment CD86 expression (Figure 4(b)). Among the scavenger, M2 receptors, Dectin-1 showed a possible oxygen-mediated effect following PAO1 exposure, as the fold change of MFI expression was highest on MH-S cells exposed to CS and 40% oxygen; in contrast, MMR was not different between the groups. Using FACS to assess PAO1-GFP association with MH-S cells, we observed a strong correlation between Dectin-1 MFI and PAO1-GFP MFI (*R* ^2^ = 0.7905) (Figure 4(c)). This data suggests that cells expressing higher levels of Dectin-1 also had higher bacterial association and also supports that 40% oxygen was an important modifier of both markers. In contrast, the correlation between CD86 MFI and PAO1-GFP MFI or MMR MFI and PAO1-GFP MFI was not nearly as strong.

### 3.6. 40% Oxygen Regulates Expression of Other Macrophage Phagocytic Receptors

**Figure 5:**
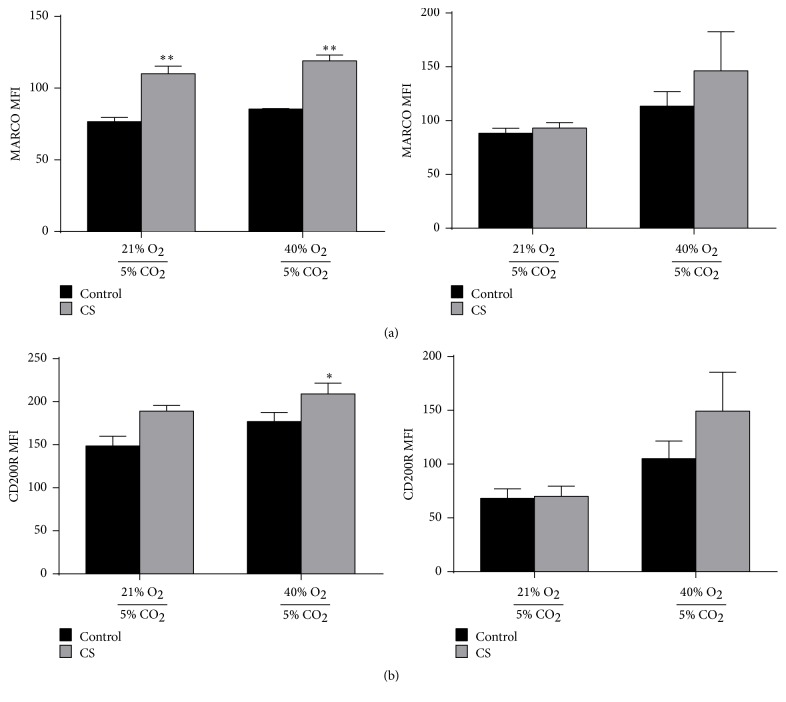
Oxygen regulates additional phagocytic receptors on CS-exposed macrophages. (a) We quantified MFI of MARCO expression on MH-S macrophages following CS and oxygen exposure prior to (left) and after (right) incubation with PAO1 bacteria (mean ± SEM, *n* = 3, ^*∗∗*^ *p* < 0.01 compared to both control + 21% O_2_ and control + 40% O_2_ groups by one-way ANOVA with Bonferroni correction). (b) We also quantified CD200R expression by MFI on MH-S macrophages prior to (left) and after (right) incubation with PAO1 bacteria (mean ± SEM, *n* = 3, ^*∗*^ *p* < 0.05 compared to control + 21% O_2_ group by one-way ANOVA with Bonferroni correction).

We also measured oxygen-induced effects on other macrophage surface receptors including MARCO and CD200R. CS-exposed MH-S cells demonstrated increased expression of MARCO; 40% oxygen did not further regulate MARCO expression (Figure 5(a)). Following subsequent incubation with PAO1-GFP, MARCO expression was not statistically different between groups, although trended towards an increase on macrophages exposed to CS + 40% oxygen. In contrast, the combination of cigarette smoke and 40% oxygen exposure significantly increased CD200R expression (Figure 5(b)) compared to control and 21% oxygen. Following incubation with PAO1-GFP, however, macrophages exposed to CS + 40% oxygen did not significantly increase CD200 expression but did trend in that direction. Collectively, this data suggests that low-dose oxygen can regulate expression of multiple scavenger or inhibitory receptors that may be important for clearance of bacteria and other immune-mediated functions.

## 4. Discussion

In this study, we sought to understand how oxygen therapy may modulate cigarette smoke-induced immune dysfunction by using an experimental model of cigarette smoke exposure and intrapulmonary inoculation with* Pseudomonas aeruginosa*, followed by room air or low-dose oxygen.* Pseudomonas aeruginosa* is an important respiratory pathogen in patients with lung disease, including COPD [22]. The addition of low-dose oxygen (FiO_2_ = 0.35–0.40) to cigarette smoke-exposed mice improved clearance of* Pseudomonas aeruginosa* leading to a reduction in mortality and measureable differences in lung injury. Phenotypically, macrophages derived from these mice upregulated pattern recognition and scavenger receptors. In cell culture, short-term exposure to 40% oxygen following cigarette smoke enhanced macrophage clearance of PAO1. Along with supportive data regarding macrophage phenotyping and cytokine production, our findings in cell culture solidify evidence for a direct effect of low-dose oxygen to improve macrophage function. However, the variable effects of 40% oxygen on macrophage surface receptors highlight potentially diverse regulation of phagocytosis.

Our study identifies a novel, protective role for low-dose oxygen to enhance macrophage phagocytosis of bacteria and mitigate lung inflammation following a subacute duration of CS exposure. Although the murine model of subacute CS exposure followed by bacterial challenge may be a reasonable model for some of the inflammatory changes seen with human bronchitis, it does not account for changes related to mucus production or for structural changes seen with emphysema. If similar benefits of low-dose oxygen on promoting bacterial clearance were found in humans with COPD or other smoking-related lung diseases, these findings may provide some basis on the benefit of oxygen therapy for patients with smoking-related lung disease during pathogen-induced disease exacerbation [1, 2]. In that context, low-dose oxygen therapy may also limit disease progression by enhancing macrophage phagocytosis and thereby limiting the severity of bacteria-induced disease exacerbations [23].

There are limited studies analyzing the immunomodulatory effects of supplemental oxygen. Most prior studies demonstrate a detrimental effect of high oxygen levels (60–100%) on lung immunity. Our findings somewhat contrast the work of Baleeiro and colleagues who demonstrated a detrimental effect of sublethal oxygen exposure on host defense against gram-negative pneumonia [5]. However, important differences in study design include the level of oxygen exposure (95% versus 40%) and the use of cigarette smoke. Their work identified a reduction in macrophage toll-like receptor 4 (TLR4) expression following 95% oxygen exposure resulting in impaired recognition of gram-negative bacteria. However, in our study, with the addition of 40% oxygen following CS exposure, we did not observe differences in macrophage surface TLR4 expression by flow cytometry (unpublished observations). We have also demonstrated that moderate levels of oxygen exposure (60%) ~12 hours after LPS-induced lung inflammation were sufficient to significantly exacerbate lung damage [6]. In one of the few studies of host defense at lower levels of supplemental oxygen, Knighton and colleagues showed that 45% oxygen decreased tissue necrosis and increased bacterial clearance compared to 12% oxygen following skin infection with* E. coli* in Guinea pigs [24]. Therefore, in addition to the impact of cigarette smoke, these studies also suggest that oxygen-induced effects on lung immunity may be dependent on the level of oxygen exposure.

We noted marked changes in macrophage cell surface phagocytic and scavenger receptor expression in response to cigarette smoke and 40% oxygen. Interestingly, Dectin-1 expression was increased following low-dose oxygen exposure in CS-exposed macrophages, and this increase strongly correlated with increased PAO1 uptake by macrophages. Dectin-1 is a type II transmembrane receptor involved in *β*-glucan-derived fungal pathogen immune responses [25, 26]. Macrophage Dectin-1 expression has been shown to be regulated by leukotriene B4 as a part of the GM-CSF/PU1.1 axis [27], and leukotriene B4 levels were elevated in human BAL fluid following exposure to 50% oxygen [28]. Dectin-1 is critical for clearance of fungal pathogens via recognition of beta-glucans [26, 29, 30]. However, beta-glucans are present in the cell walls of other nonfungal pathogens including* P. aeruginosa* [31, 32] and* H. influenza*; for the latter, beta-glucan recognition by epithelial Dectin-1 was critical to generate an inflammatory response [33, 34]. With the strong correlation between macrophage Dectin-1 expression and PAO1 phagocytosis among MH-S macrophages in culture, oxygen-induced upregulation of Dectin-1 may be contributory towards clearance of PAO1 via beta-glucan recognition and binding.

We suggest that oxygen improves bacterial phagocytosis through upregulation of cell surface phagocytic and scavenger receptors. However, there are other possible mechanisms for the protective effects of oxygen. One consideration is that room-air-exposed mice were more hypoxic following bacteria exposure and supplemental oxygen may have protected the mice from hypoxia-induced complications. We did not assess for hypoxia* in vivo*, but we did demonstrate a significant increase in macrophage phagocytosis of bacteria with low-dose oxygen exposure in our cell culture system where hypoxia was not a factor. Another potential mechanism by which low-dose supplemental oxygen may improve bacterial phagocytosis is through enhanced production of reactive oxygen species (ROS). NADPH oxidase, a key enzyme in macrophages for generation of superoxide that requires molecular oxygen, modulated bacterial overgrowth [35]. However, unregulated ROS can also be detrimental to bacterial control, as a loss of macrophage-generated extracellular superoxide dismutase (EC-SOD), an antioxidant enzyme, was found to impair phagocytosis of* E. coli* [36]. Interestingly, hyperoxia (100% oxygen) in EC-SOD knockout mice leads to increased lung edema and diminished survival [37], a further evidence that unopposed ROS may augment lung damage. Overall, our findings suggest that oxygen-induced modulation of macrophage function is complex and likely associated with both the underlying lung substrate and the level of supplemental oxygen that is administered.

Our study also highlights the complex regulation of cell surface signaling receptors that is influenced by supplemental oxygen. To our knowledge, this is the first report of macrophage CD200R expression in response to oxygen and cigarette smoke exposure. CD200R demonstrates an inhibitory effect on inflammatory signaling when engaged by the CD200 ligand that is expressed by the respiratory epithelium and other immunomodulating cells (e.g., T cells) [7]. Prior studies have shown decreased expression of CD200R in human monocytes after treatment with diesel emission particles [38]. In contrast, we showed that the combination of cigarette smoke and 40% oxygen exposure increased alveolar macrophage CD200R expression. CD200R may protect against excess CS- and bacteria-induced damage, as it did in a murine influenza model [8]. In addition, due to the important immunomodulatory effects of macrophage CD200R tethering to epithelial CD200, we would anticipate a synergistic effect in terms of limiting inflammation in a macrophage-epithelial coculture system.

Our study has a few limitations. One, because we did not directly measure bacterial killing by macrophages, it is conceivable that low-dose oxygen exposure did not enhance phagocytosis, but instead impaired bacterial killing. If true, we would not anticipate a benefit of low-dose oxygen on murine mortality and lung inflammation. Two, we did not evaluate for an oxygen-mediated effect on neutrophil clearance of bacteria. Although neutrophil numbers in the alveolar space did not appear to be influenced by oxygen following CS and PAO1 exposure, we did not directly assess neutrophil function. Macrophage CD86 and Dectin-1, both modulated by CS and oxygen exposure in our studies, can promote neutrophil costimulation [39] and pathogen clearance [40]. However, since low-dose oxygen-enhanced macrophage phagocytosis of bacteria in isolated macrophages in culture, any oxygen-mediated effects on neutrophils would likely enhance, not diminish, our findings. Three, although the* in vitro* model for CS delivery induced IL-6 secretion by alveolar macrophages, we did not observe a reduction in PAO1 phagocytosis amongst CS-exposed MH-S cells as compared to control exposure. However, prior study of cell culture systems investigating bacteria phagocytosis have primarily used cigarette smoke extract (CSE) and not direct cigarette smoke exposure [20, 41, 42]; thus little is known about what duration and intensity of direct CS exposure to macrophages are required to induce a change in phagocytosis. Four, because we have not proven that upregulation of one or several scavenger receptors is critical for low-dose oxygen to enhance macrophage phagocytosis, it is possible that receptor upregulation is correlative and not causal for oxygen-enhanced phagocytosis. Given the concomitant use of diverse pattern recognition receptors including Dectin-1, MMR, and the TLRs by immune cells, we would anticipate synergy between these receptors towards antimicrobial immunity [43]. Our results are similar to that of Hodge and colleagues who demonstrated a clear benefit of azithromycin exposure on human alveolar macrophage phagocytosis with a correlative increase in MMR expression, yet specific cellular mechanisms were not ascertained [44].

## 5. Conclusion

Our findings demonstrate a novel, protective role for low-dose oxygen in cigarette smoke and bacteria-exposed mice that appears to be mediated by enhanced macrophage phagocytosis of bacteria. Upregulation of scavenger and other pattern recognition receptors also denotes a unique cellular phenotype with coinduction of M1 and M2 macrophage markers. The individual role of these receptors is not yet clear, but our work has identified additional potential therapeutic targets to support enhanced bacterial clearance and decreased inflammation seen in response to 35–40% oxygen treatment in the lungs of cigarette-smoke-exposed mice.
